# Behavioral Reluctance in Adopting Open Access Publishing: Insights From a Goal-Directed Perspective

**DOI:** 10.3389/fpsyg.2021.649915

**Published:** 2021-04-07

**Authors:** Massimo Köster, Agnes Moors, Jan De Houwer, Tony Ross-Hellauer, Inge Van Nieuwerburgh, Frederick Verbruggen

**Affiliations:** ^1^Research Group of Quantitative Psychology and Individual Differences, KU Leuven, Leuven, Belgium; ^2^Center for Social and Cultural Psychology, KU Leuven, Leuven, Belgium; ^3^Department of Experimental Clinical and Health Psychology, Ghent University, Ghent, Belgium; ^4^Open and Reproducible Research Group, Graz University of Technology and Know-Center GmbH, Graz, Austria; ^5^Ghent University Library, Ghent University, Ghent, Belgium; ^6^Department of Experimental Psychology, Ghent University, Ghent, Belgium

**Keywords:** open access publishing, behavioral reluctance, goal-directed, meta-research, intervention

## Abstract

Despite growing awareness of the benefits of large-scale open access publishing, individual researchers seem reluctant to adopt this behavior, thereby slowing down the evolution toward a new scientific culture. We outline and apply a goal-directed framework of behavior causation to shed light on this type of behavioral reluctance and to organize and suggest possible intervention strategies. The framework explains behavior as the result of a cycle of events starting with the detection of a discrepancy between a goal and a *status quo* and the selection of behavior to reduce this discrepancy. We list various factors that may hinder this cycle and thus contribute to behavioral reluctance. After that, we highlight potential remedies to address each of the identified barriers. We thereby hope to point out new ways to think about behavioral reluctances in general, and in relation to open access publishing in particular.

## Introduction

We often seem to know that we should change our behavior, but often we do not. Such behavioral reluctance is impeding much needed change in various types of behavior. One such type of behavior is academic open access publishing. Open access journals receive more and more attention because there is a growing awareness of drawbacks associated with traditional, non-open access journals (e.g., limited dissemination, copyright issues, high subscription costs for libraries, and inefficient use of taxpayer’s money; e.g., Xia, 2010), while good reasons to adopt open access journals are being brought forward (e.g., more citations, increased media coverage, and retaining author rights; for a review of such benefits, see, e.g., McKiernan et al., 2016). Research reveals an increasing awareness of open access through time (Xia, 2010) and most researchers seem to be in favor of the general concept. Studies have, for instance, found that between 80 and 90% of researchers reported having positive attitudes toward open access publishing (Dallmeier-Tiessen et al., 2011; Davis, 2014; Ross-Hellauer et al., 2017).

However, despite endorsing the benefits, many scholars seem hesitant to reorient and publish their own works in open access journals (Nelson, 2009; Edwards, 2016; Johnson, 2018). In one landmark study, positive attitudes among almost 90% of respondents translated into only 52% who said they had already published in open access journals (Dallmeier-Tiessen et al., 2011). Although the dissociation between attitudes and behavior may have shrunk today, it has far from disappeared (Rowley et al., 2017; O’Hanlon et al., 2020). Thus, despite the fact that researchers are increasingly aware of open access, and seem to value it in the abstract, they are still reticent to put it into practice, resulting in a so-called attitude-behavior gap.

**Figure 1:**
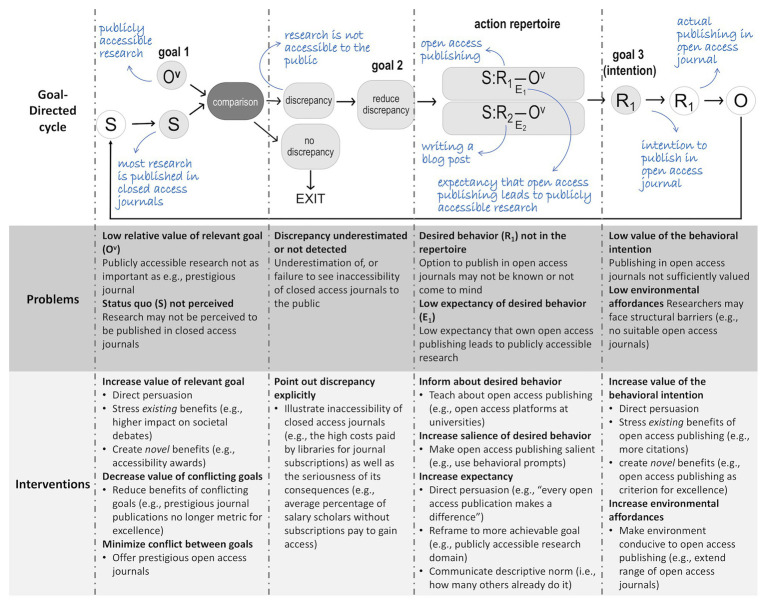
The goal-directed cycle: problems and interventions in open access publishing. The general goal-directed cycle is depicted with white bubbles as observable factors; gray bubbles with black letters as mental representations; gray bubble with white letters as a mental operation. S, *status quo* (or stimulus); O^v^, valued outcome (V = value); O, actual outcome; E, expectancy; R, response or behavior. The application of the goal-directed cycle to open access publishing is depicted in blue handwriting. Problems and intervention strategies are located underneath the goal-directed cycle according to where they become relevant in the cycle. Figure adapted from Moors et al. (2019).

In this paper, we will use a goal-directed framework of behavior causation proposed by Moors et al. (2017) and Moors (2019) to shed light on this issue. We think this framework proves particularly useful to understand the underlying causes of behavior and can thereby help to (a) clarify the attitude-behavior gap in open access publishing and (b) derive ideas for how researchers can be encouraged to actually adopt open access publishing. In a first section, we describe how the sequence of events in the goal-directed framework (which is discussed in greater detail in Box 1) can be applied to open access publishing. In a second section, we discuss various factors that may impede the behavioral cycle and how these factors may contribute to a reluctance to submit papers to open access journals. The discussion of each factor will be followed by a proposal of intervention strategies. For an illustration of the goal-directed framework, the identified barriers, and the proposed intervention strategies, see **Figure 1**. We thereby hope to provide theoretical insights into behavioral reluctances in general and in relation to open access publishing in particular as well as to offer new potential avenues for more effective behavior change to promote open access publishing.

Box 1 The goal-directed framework.

The goal-directed framework proposes that most behavior can be explained by the following cycle of events (see Figure 1, adapted from Moors et al., 2019): A perceived status quo (i.e., the representation of a stimulus; S) is compared to a first goal (i.e., the representation of a valued outcome; Ov). When a discrepancy between the stimulus and the first goal is perceived, a second goal arises, which is to reduce the discrepancy. This can be achieved with one of the following three strategies. The person might (a) change the stimulus by acting (i.e., assimilation), (b) change the first goal (i.e., accommodation), or (c) change the interpretation of the stimulus (i.e., immunization). To illustrate, imagine you put a lot of effort into cooking a nice dinner for your friend and the friend says that the food does not taste good (stimulus), which leads to a discrepancy with your goal to be seen as a good cook. In response, you may try to change the dish until your friend likes it (i.e., assimilation), you may give up on the goal to be a good cook (i.e., accommodation) or you may convince yourself that your friend has a strange taste and that it does not reflect how good the food actually is (i.e., immunization). Which of these three strategies is chosen likely depends on how useful they are in reducing the discrepancy. The usefulness of a strategy or action is referred to as its expected utility and depends on the value of the outcome of having the discrepancy reduced and the expectancy that the strategy will indeed lead to such a reduction. If a person chooses to act (i.e., assimilation), they still need to choose a specific action option from their action repertoire. If the action repertoire contains more than one action option, the expected utilities of these options are compared and the option with the highest expected utility should be chosen. Again, the expected utility of one action option depends on the value of the outcome and the person’s expectancy that the action will indeed lead to this outcome. Once a specific action option has been chosen, the corresponding intention to engage in the action (i.e., action tendency) is activated. This intention can be considered as a third goal and will translate into an overt action. Once the action is performed, it is followed by an actual outcome, which may or may not constitute a change in the status quo (i.e., the stimulus). This outcome is then fed back as the input to a new cycle. The cycle is repeated until the discrepancy is effectively removed.

The goal-directed model further assumes that organisms have multiple goals and that these are organized in a goal hierarchy. This entails that cycles for subordinate goals are embedded into cycles for superordinate goals. The same principles that guide the selection of an intention to act in order to reach a subordinate goal also guide the selection of a subordinate goal in order to reach a superordinate goal. A person chooses a subordinate goal because it has a high expected utility to satisfy a superordinate goal. Accordingly, the value of a subordinate goal reflects its expected utility to satisfy one or more superordinate goals.

### The Goal-Directed Framework Applied to Open Access Publishing

Applying the goal-directed framework outlined in Box 1 to the case of open access publishing, the perceived *status quo* might be that research is not accessible to the public but rather hidden behind a paywall by private publishers. Researchers may hold a number of different goals that may serve as reasons for open access publishing. The key perceived benefits of open access publishing, reported in a meta-analysis of other studies (Togia and Korobili, 2014), were improved access to research for the general public and academics in less wealthy universities, wider diffusion of research outputs, increased citation and impact, faster publication times, and reduced costs. Another benefit might be that it gives researchers more control over the publication process (thereby changing the traditional publication model). Each of them could be taken as the first goal that starts off the cycle. For the sake of clarity, we will start from the goal to make research more accessible to the public. However, the same exercise could be repeated with other goals in mind.

As long as researchers perceive a discrepancy between the *status quo* and the first goal to make research accessible to the public, they develop the second goal to reduce this discrepancy, either by (a) taking overt action to make research accessible to the public (i.e., assimilation), (b) changing this goal or reducing its value (i.e., accommodation), or (c) ignoring that research is not accessible to the public or by reassuring themselves that the media will communicate research findings from closed journals to the public (i.e., immunization). Which of these three strategies is chosen likely depends on respective expected utilities of these strategies for reducing the discrepancy. If researchers choose to engage in overt action (i.e., assimilation), they still need to select the action option from their action repertoire with the highest expected utility. Once researchers have chosen the action option they deem most effective to make their research accessible to the public, the corresponding intention is activated and is then translated into an overt action. If researchers, for instance, estimate that publishing in open access journals has a higher expectancy of making their work accessible than writing a blog post, the intention to publish in open access journals is activated. After this intention is translated in the actual publishing in open access journals, it is followed by an outcome. The outcome is fed back as the input to a new cycle and this cycle is repeated until the discrepancy is effectively removed. Researchers may thus continue to publish in open access journals until their research is sufficiently accessible to the public. Furthermore, as discussed in Box 1, our framework postulates a hierarchical goal structure. This implies that the value of the goal to make one’s research accessible to the public corresponds to the expected utility that fulfillment of the goal will be conducive to a number of superordinate goals such as the goals to increase the visibility of one’s research, to have impact on societal debates, or to foster collaboration among researchers.

From this perspective, it becomes clear that an attempt to find an explanation for the occurrence of the behavior of open access publishing is an attempt to explain the result of a complex chain of events. Understanding why there is a reluctance to engage open access publishing then comes down to understanding how and why the sequence leading up to this behavior might be hindered. As the reluctance to publish in open access journals impedes an important change in the transition toward more accessible and transparent research practices, there is a growing interest in understanding the potential barriers as well as avenues for a large-scale adoption of open access publishing (e.g., Björk, 2004). We will, therefore, move on to consider factors that may hamper the goal-directed cycle that should give way to open access publishing and thereby hope to offer new insights into why the reluctance among researchers to embrace open access publishing is so tenacious.

### Factors That May Impede the Goal-Directed Cycle in the Context of Open Access Publishing

#### Values of Goals

A first factor is the value of goals relative to that of other goals. Researchers may be reluctant to adopt a behavior that can reduce the discrepancy between the *status quo* and one goal because they expect the behavior will itself be discrepant with another goal that has a higher value. It is, therefore, important to consider the values that researchers ascribe to certain goals relative to those of other goals. Researchers may, for instance, not submit their paper to an open access journal, despite the fact that this is instrumental for their goal to make their research accessible to the public, if doing so also constitutes a discrepancy with or has a low expectancy to reach the more important goals to communicate with peer researchers from the same research area or to collect publications in prestigious journals marked by high impact factors (Xia, 2010). That the latter may turn into a barrier is illustrated by a Canadian Science Publishing survey on researcher’s priorities in selecting publishing options, which found that the availability of open access options ranked only 6th out of 18 criteria when selecting a journal and was eight times less important to researchers than a journal’s impact factor and 13 times less important than journal reputation (Davis, 2014). In a similar vein, a global survey conducted by Nature Publishing Group and Palgrave Macmillan found that the option to publish open access ranked only 14th out of 17 criteria, with the most important factor listed as journal reputation (Nature Publishing Group/Palgrave Macmillan, 2015).

**Figure 2:**
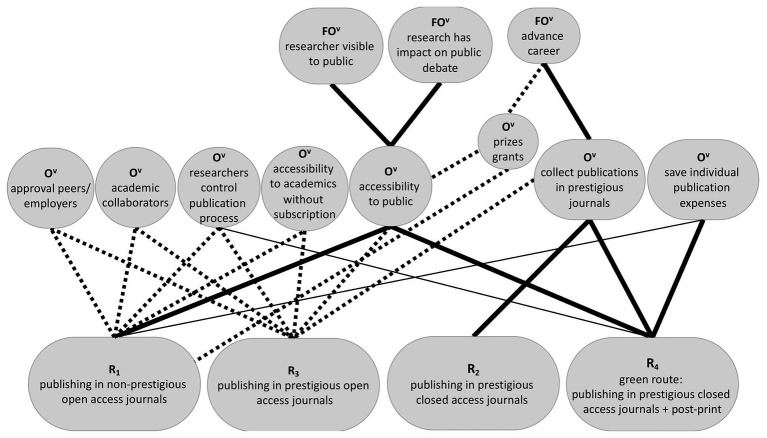
Exemplary goal-hierarchy involved in open access publishing. Thick full lines represent high expectancies of subordinate goals to superordinate goals. Thin full lines represent weak or negative expectancies of subordinate goals to superordinate goals. Thick dotted lines represent interventions that might establish high expectancies of subordinate goals to superordinate goals. R, response or behavior; O^v^, valued outcome (V = value); and FO^v^, further valued outcome.

To address this, at least three types of interventions can be suggested. One might try to (a) increase the value researchers ascribe to the goal of making research accessible to the public, (b) decrease the value of other, competing goals such as collecting publications in prestigious journals, or (c) change the environment so that these goals no longer conflict with each other, for instance, by creating prestigious (high-quality) open access journals. We discuss these options in turn alongside an exemplary goal-hierarchy involved in open access publishing (see Figure 2).

Increasing the value of the goal to make research accessible to the public may be done by directly underscoring the importance of this goal, or indirectly, by pointing out that this goal is instrumental for superordinate goals such as increased visibility of the researcher to the public, increased impact on societal debates and policy making, and increased collaboration among researchers. In addition to pointing out *existing* links between the subordinate goal to make research publicly accessible and superordinate goals, one might also create *novel* links between this subordinate goal and superordinate goals (i.e., add further benefits) by implementing changes in the reward structure of the environment. To stimulate researchers to endorse the goal of public accessibility, universities could, for instance, lend accessibility awards to researchers who make significant efforts to promote the accessibility of their research. Another option could be to make accessibility an important quality criterion for the allocation of research grants (an option that is already taken by certain funding agencies). In these cases, accessibility of research would become instrumental to superordinate goals (gaining awards and research grants).

An alternative strategy is to decrease the value of goals that hinder adopting open access publishing, such as the goal to collect publications in prestigious journals, the goal to save publishing expenses for the individual researcher, and the goal to save time and energy involved in the administrative payment process of publication fees. This can be done by minimizing the values of these other goals directly, or indirectly by pointing out or making sure that these other goals are no longer instrumental for other superordinate goals. To illustrate the indirect pathways, researchers could be convinced that publications in prestigious journals alone do not reflect the quality of one’s work, but better still, universities and funding agencies could attach less weight to publications in such prestigious journals. This would be in line with Alperin et al. (2019) suggestion to attach more weight instead to the degree to which research serves the public good in quality assessments, which entails public access.

As it may be difficult to convince researchers of the relativity of publications in prestigious journals and even to fully justify it, the best solution may be to change the reward structure of the environment in such a way that the goals of public accessibility and of publications in prestigious journals no longer conflict with one another. This can be done by creating (or facilitating the emergence of) prestigious open access journals. Note, moreover, that the prestige or quality of journals may better be captured by other metrics than impact factors, a suggestion that received support in the Declaration on Research Assessment (DORA, sfdora.org). In a similar vein, to reduce the value of the goals to save on individual publishing expenses and on time spent on administrative payment procedures, universities could offer to pay the fees for open access publishing (a measure already adopted by several universities) and to fully take care of the administrative aspect, for instance, by setting up contractual agreements with journals to cover publication fees. This is increasingly realized in the form of transformative agreements, which seek to move the stake of contracts between institutions and publishers away from subscription-based reading toward open access publications. While transformative agreements could help to reduce the administrative burden of the payment procedure for researchers, they have also been criticized for the fact that they do not sufficiently change the publishing model, resulting in a costly way of achieving open access (e.g., ESAC, 2018; Lawson, 2019; Olsson et al., 2020). A better solution might be, therefore, to establish more open access journals that do not charge authors processing fees, so-called diamond or platinum open access journals.

Considering that the intention to engage in a behavior is also a goal, one can also use the aforementioned strategies to increase the value of the behavior to publish in open access journals itself and to decrease the value of alternative behaviors. Increasing the value of open access publishing could be done directly by communicating about the importance of open access publishing. In a more indirect way, one could also point out *existing* benefits of open access publishing besides increased public accessibility (and not mediated by increased public access). For instance, one could point out that open access publishing helps researchers take back control over the publication process, attracts more potential academic collaborators, increases citations, and does already lead to increased job and funding opportunities in some universities (see McKiernan et al., 2016). One could also indicate that the goal to publish in open access journals is instrumental to the goal of gaining approval among peers. This comes close to an effective intervention strategy known as the provision of injunctive norms (Farrow et al., 2017). Such a norm informs people about what others consider important and hence what is likely to gain their approval. Evidence for the usefulness of injunctive norms in open access publishing comes from a study by Moksness and Olsen (2017), who found that the perception of an injunctive norm that others value open access publishing, predicted an increase in the intentions to publish in open access journals. To give an example of how an injunctive norm may be communicated, researchers could be informed that colleagues in their research group find it important to publish in open access journals or platforms, for instance, by making it part of the mission statement on the research group’s website. Another option would be to create *novel* benefits of the behavior of publishing in open access journals (again, not mediated by the goal to increase public access) by implementing changes in the reward structure of the environment. For instance, universities can list open access publishing as a criterion for excellence or research integrity.

Reducing the value of alternative behaviors can likewise be done in direct and indirect ways. For instance, researchers may have selected the behavior to publish in a closed access journal but to self-archive the paper and make a post-print available (i.e., the “green route”) because it is instrumental for the goal of public access and at the same time, avoids publication fees for individual researchers as well as their administrative burden with the payment process. In addition to directly communicating a low value of this alternative behavior, one can indirectly influence its value by pointing out that the green route does not contribute to other goals in the way that open access publishing does and that it may have certain costs. One could, for instance, highlight uncertainty about copyright/licensing issues and the variety of publisher self-archiving policies, which seem to be major barriers to self-archiving (Hoorn and van der Graaf, 2006; Gadd et al., 2007; Gulley, 2013; Spezi et al., 2013). Another downside that may be pointed out is that continuing to publish in closed access journals (in combination with the green route) does nothing to increase researcher’s control over the publication process.

#### Discrepancy Detection

A second factor that might impede behavior is when a person fails to detect a discrepancy between the *status quo* and their goal or when they underestimate the magnitude of this discrepancy. For instance, researchers may be aware that traditional journals can only be accessed by a paid subscription and may have the goal to make research accessible to the public but fail to realize that the paywall is discrepant with accessibility to the public and hence fail to activate the goal to do something about it. A potential solution could be to point out the discrepancy explicitly. This could be done by illustrating how restricted the access to traditional journals is (e.g., how immense the fees are that libraries have to pay for subscriptions to traditional journals or how small of a percentage of the world population has subscription-based access) and how serious the consequences are for various groups of potential readers (e.g., how much personal salary scholars from less wealthy universities spend on average to access research in traditional journals).

#### Action Repertoire

A third factor that may account for behavioral reluctance is a lack of suitable response options in a person’s action repertoire. Researchers may detect a discrepancy between publishing in traditional, closed access journals and their goal to make research accessible to the public, but the response option to publish in open access journals might not come to mind. With the majority of research still being published in traditional closed access journals, it can be assumed that there is not yet a culture of considering open access journals. The perceived lack of this response option may result in a low expected utility of assimilation relative to accommodation and immunization. As the latter two strategies do not involve personal actions to reach the goal of making research more accessible, choosing to engage in these strategies amounts to surrendering oneself to behavioral reluctance and thus to the continuation of publishing in closed access journals. To facilitate behavior in these cases, one could try to extend a person’s action repertoire by teaching them about the option of open access publishing, for instance, *via* online directories that index and provide access to high quality, peer-reviewed open access journals (e.g., doaj.org) or *via* open access platforms at universities, which increase awareness and provide researchers with practical information (e.g., see recently established *Open Science Platform*; KU Leuven, 2020). For researchers who are already informed about the option of open access publishing, the option may still need to be made accessible in the appropriate contexts. This could be done, for instance, by using behavioral prompts, which serve as behavioral reminders (Sussman et al., 2013). Behavioral prompts can be implemented, for instance, *via* a logo that represents open access publishing and that pops up when researchers make science-related internet searches.

It is of course still possible that open access publishing is hindered by an actual lack of concrete response options to implement the intention to publish in open access journals. It is, therefore, also important to consider the affordances of an environment with regard to the behavior in question. If the environment does not allow researchers to publish their work in open access journals despite the intention to do so, a structural change of the environment may be necessary. For instance, researchers might have the intention to publish in open access journals but be confronted with a lack of open access journals that are suitable for their research domain (Kling and McKim, 2000). Addressing such barriers would require structural changes, such as an extension of the range of open access journals to cater for all research domains.

#### Expectancies

A fourth factor that may lead to behavioral reluctance is that people’s expectancies are not always accurate. Researchers might, for instance, have a low expectancy that publishing in open access journals will increase the public accessibility of their research, making assimilation less likely. They might even estimate the impact of publishing in open access journals to be so negligible in reaching their goal to make research accessible to the public, that they give up this goal (i.e., accommodation). They might also reassure themselves that the media will communicate the findings of their research to the public and that this is more effective than publishing in open access journals (i.e., immunization).

Open access publishing can be considered as a collective action, in which the actions of many are required to achieve a large-scale outcome. To achieve a transition toward publicly accessible science, a behavior change by a large collective of researchers is required. In such situations, a single person might feel incapable to reach the outcome with her own actions (i.e., low expectancy in assimilation) and thus refrain from engaging in the behavior. An intervention to facilitate behavior change in these cases might be to change expectancies directly, for example, by setting up a campaign to stress that every single publication in an open access journal compared to a publication in a closed journal makes a meaningful difference in making research accessible to the public. More indirect ways to change expectancies are to alter the framing of the goal so that it seems more achievable by a single individual. One could, for example, reframe the goal from making science accessible to the public to making research in a certain domain, such as neuroscience, accessible to the public. Alternatively, one could reframe the goal from making science accessible to the public in general to making it accessible to a specific group, such as researchers in developing countries, alumni, or professionals. By framing the goal with regard to one’s own domain and/or with regard to a certain group, it may appear more achievable. Another indirect way to change expectancies would be to stress how many others are already publishing in open access journals, thereby increasing the expectancy that an individual researcher’s work will make a difference in the transition toward more accessible research. The latter strategy has already been shown to be effective across many behavioral domains and is referred to as the provision of descriptive norms (Cialdini, 2007; Farrow et al., 2017). There is also initial evidence showing that the perception of a descriptive norm about others publishing in open access journals predicts researchers’ own intentions to engage in open access publishing (Moksness and Olsen, 2017). To give an example how a descriptive norm may be communicated, institutions or research groups could showcase the number of realized open access publications to their members.

#### Representational Quality

A factor that runs through all previous ones is the quality of the representations involved in the goal-directed cycle (Moors, 2015). If one or more of these representations is not sufficiently activated, they cannot play their role, and the cycle may not be initiated, may be aborted, or may be altered. For instance, the representations of stimuli and valued outcomes will not enter in comparison as long as one or both of them lack sufficient representational quality. Researchers may fail to notice that a large part of society has no access to their research if it is published in a traditional journal. They may also not always consider their goal to make research publicly accessible. This helps to explain why, in some instances, researchers may fail to notice a discrepancy between a stimulus and a goal (see Factor 2). Similarly, the representational quality of a specific action option, such as to publish in open access journals, may be too weak to be considered in the action repertoire. Researchers may have knowledge that publishing in open access journals has a high expectancy to lead to the valued outcome of making their research accessible to the public, but if the option to publish in an open action journal is not on their mind (i.e., the representation of the action option lacks sufficient activation), they are unlikely to select it.

To facilitate behavior change at this level requires facilitating the processing of the relevant stimuli, goals, and/or action options. This can be achieved, for instance, by increasing the frequency (e.g., *via* repetition) or recency (e.g., *via* priming, i.e., recent pre-activation) of the relevant representations or by drawing attention to them. To foster open access publishing, one could, for instance, repeatedly expose researchers to current accessibility restrictions or increase the recency of exposure to the goal to make research accessible. In particular, one could expose researchers regularly to illustrations of limited access and prompt them with the goal to make research accessible prior to an upcoming publication. This could be done, for instance, by marking closed access publications with a closed access sign. This is in line with the intervention strategy of mental contrasting proposed by Oettingen (1999). Mental contrasting also involves directing attention to the *status quo* and the goal, and is considered the best way to increase salience of the discrepancy between these two elements and to mobilize a person to take action. Finally, if an action option is not sufficiently activated to be accessible for being chosen, the representation of the desired action option (i.e., publishing in open access journals) may be facilitated *via* behavioral prompts (e.g., Sussman et al., 2013).

## Discussion

Many scholars seem reluctant to publish in open access journals despite recognizing that it is an indispensable way to make their research more broadly accessible. In this paper, we explained this behavioral reluctance making use of the goal-directed framework by Moors et al. (2017) and Moors (2019). In this framework, behavioral reluctance can be understood as the result of problems in a cycle of events in which the detection of a discrepancy between a *status quo* and a goal is alternated with the selection of strategies to resolve this discrepancy. We identified problems related to values of goals, discrepancy detection, action repertoire, expectancies, and representational quality. For each of the problems discussed, we also discussed various openings for behavior facilitation. Among the potential interventions that we listed, some were situated at the level of the individual, whereas others were more structural in nature. On the individual level, we clarified how the values of people’s goals, their beliefs, and the quality of certain representations can be targeted to remedy for behavioral reluctances. On the structural level, we highlighted the need to change the reward structure of the environment so that it is less likely to probe conflicting goals as well as the need to change the affordances of the environment to facilitate behavior once the intention to engage in the behavior has been formed. In the long run, it would be important to monitor and evaluate the effectiveness of these proposed intervention strategies for changing publishing behavior. This will be an important avenue for future research.

Overall, we hope that following these different pathways would not only serve as immediate remedies to resolve individual reluctances, but that they can help to establish and solidify a stable behavioral culture. If certain behaviors become part of a culture, it is likely that a self-perpetuating effect occurs because most of the highlighted pathways should be naturally fostered in such a culture. If open access publishing becomes an integral part of the culture in universities, researchers would be more likely to be exposed to the importance of the goals of open access publishing (e.g., the accessibility of research) as well as of different superordinate goals that favor open access publishing (e.g., that others approve of open access publishing or that it can help to increase visibility and inform societal debates). In addition to getting acquainted with an injunctive norm that favors open access publishing, researchers would also be more likely to be exposed to a descriptive norm that favors such a practice because if more scholars in their immediate surroundings already publish in open access journals, engaging in the same behavior would appear more effective to each individual researcher as a means to make research accessible. This may be one potential explanation for the finding that when descriptive and injunctive norms dissociate, people tend to follow the descriptive norm rather than the injunctive norm (Farrow et al., 2017). Finally, in a culture that endorses open access publishing, researchers might be regularly exposed to the goal to make research accessible and to the action option to publish in open access journals.

It may also be worthwhile to situate open access publishing in its broader sociological context. In 1942, the sociologist Merton (1973/1942) proposed that the scientific system was governed by four broad norms: communism, universalism, disinterestedness, and organized skepticism. These norms can be regarded as broad injunctive norms (i.e., what others find important) of the scientific community because they specify the goals that are valued by the scientific community. For instance, “communism” encourages the goal to collaborate; “disinterestedness” encourages communal goals over the pursuit of personal interests. Today, however, most researchers seem to pursue goals that are in line with counter-norms, such as the goals to compete and prioritize self-interest (Mitroff, 1974; Anderson, 2000). As these norms describe the goals that most researchers are currently pursuing, they qualify as broad descriptive norms (i.e., what others actually do, here pursue). Here again, an exclusive focus on conveying injunctive norms may not be sufficient given the above-mentioned finding that when injunctive and descriptive norms dissociate, people tend to follow the descriptive norm (Farrow et al., 2017). The key to changing adherence to the injunctive norms may be to change the descriptive counter-norms first. Several solutions were presented in this paper for how to change researcher’s publishing behavior, and thus for how to indirectly alter the prevailing descriptive norms. For instance, one strategy could be to reconcile the collaboration/communal and competition/self-interest goals by changing the reward structure. Institutions could, for instance, seek ways in which they can make collaborative efforts an important criterion for the evaluation of researcher’s personal achievement. By doing the right thing for the wrong reasons initially, people may end up doing the right thing for the right reasons eventually. If the reward structure is changed so that researchers collaborate more to gain personal achievements initially, for instance, they may internalize the goal to collaborate and thus start to value collaboration more for its own sake.

To conclude, we hope to have illustrated that adopting the goal-directed framework to behavioral reluctance in the domain of open-access publishing offers a pragmatic path forward and helps to develop concrete intervention strategies for tackling publishing behavior that may in the long run also contribute to a wider change in the research culture. Translating the outlined intervention strategies into practical applications will still require creative work and a close consideration of the specific contexts in which an intervention takes place. The analysis at hand focused on one particular goal (i.e., to make research accessible) and on one particular group of actors (i.e., researchers) to illustrate the framework and its application. We have mentioned a number of other goals that might start off the behavioral cycle for researchers. In addition to exploring the role of other goals such as these, it will be important to explore the role of different actors and thus target groups for interventions. Different groups of actors, such as early-career compared to senior researchers, likely differ with regard to the barriers that they identify (e.g., because of differences in terms of the values they ascribe to goals, and their action repertoires and expectancies). Addressing these additional questions may help researchers and other stakeholders to deepen their understanding of the reluctance to publish in open access journals. Finally, we hope that the current exercise will pave the way for others to apply the goal-directed framework to reluctances in related domains (e.g., the reluctance to adopt open access repositories), as well as to reluctances in completely different domains (e.g., the reluctance to adopt pro-environmental behaviors).

## Author Contributions

MK and AM: writing – first drafts and revisions, development of theoretical framework, and application to open access publishing. AM: supervision. JDH, TR-H, IN, and FV: suggestions for applying the theoretical framework to open access publishing and writing – revision. All authors contributed to the article and approved the submitted version.

### Conflict of Interest

The authors declare that the research was conducted in the absence of any commercial or financial relationships that could be construed as a potential conflict of interest.
